# In Vitro Analysis of the Removability of Fractured Prosthetic Screws within Endosseous Implants Using Conventional and Mechanical Techniques

**DOI:** 10.3390/ma16237317

**Published:** 2023-11-24

**Authors:** Gisela Senent-Vicente, Mar Baixauli-López, Eva González-Angulo, Luisa Fernández-Bravo, Álvaro Zubizarreta-Macho, Miguel Gómez-Polo, Eduardo J. Selva-Otaolaurruchi, Rubén Agustín-Panadero

**Affiliations:** 1 Department of Stomatology, Faculty of Medicine and Dentistry, Universitat de València, 46010 Valencia, Spain; 2 Department of Implant Surgery, Faculty of Health Sciences, Alfonso X el Sabio University, 28691 Madrid, Spain; 3 Department of Surgery, Faculty of Medicine and Dentistry, University of Salamanca, 37008 Salamanca, Spain; 4 Department of Conservative Dentistry and Orofacial Prosthodontics, Faculty of Dentistry, Complutense University of Madrid, 28040 Madrid, Spain

**Keywords:** abutment, fractured abutment screws, dental implants, screw, internal connection implant

## Abstract

Statement of problem: The extraction of fractured abutment screws can be a difficult challenge to overcome. Purpose: To compare the removal capacity, dental implant connection damage, and time required to remove the fractured abutment screws between three drilling techniques and a conventional method. Materials and methods: A total of 180 prefabricated screw-retained abutments were intentionally fractured in internal connection dental implants after being subjected to a cyclic load and a static compression load. Afterwards, three operators randomly removed the fractured abutment screws with the following drilling techniques and a conventional method: A: a conventional technique using an exploration probe and ultrasonic appliance (n = 45), Rhein83^®^ (n = 45); B: Sanhigia^®^ (n = 45); C: Phibo^®^ (n = 45). Two-way ANOVA models were estimated to evaluate the mean time according to the method and operator used. Results: The probability of removal of the screws with mobility was twelve times higher than that of the screws without mobility (OR = 12.4; *p* < 0.001). The success rate according to the operators did not show statistically significant differences (*p* = 0.371). The location of the fractured screw did not affect removal success (*p* = 0.530). The internal thread of the implant was affected after the removal process in 9.8% of the cases. The mean extraction time was 3.17 ± 2.52 min. The Rhein83^®^ method showed a success rate of 84.4%, followed by the Phibo^®^ and conventional methods (71.1%) and the Sanhigia^®^ method (46.7%). Conclusions: The Rhein83^®^ drilling technique increases the removal probability of fractured abutment screws. The initial mobility of the fragment is also a significant factor in the removal success.

## 1. Introduction

Implants must be placed according to the requirements of the prosthetic treatment to be performed and are not based on the availability of bone [1]. With good planning, the probability of subsequent complications is reduced [2,3]. Among the most frequent complications is the loosening of the fixation screw, which despite not being a catastrophic complication and presenting with an easy solution, can often be followed by a bigger problem: the fracture of the fixation screw inside the implant [4,5,6]. Additionally, Tsuruta et al. (2018) highlighted that the abutment screw fracture may increase the gap at the implant-abutment interface, leading to the development of peri-implantitis [7].

An ideal extraction of a fractured screw inside an implant would be to extract it without damaging its internal thread, avoiding major consequences such as the extraction of the implant [8,9]; if the internal thread remains intact, the solution will only require the placement of a new screw, making it possible to reuse both the implant and the prosthesis. Katsavochristou et al. (2019) reported that the abutment screw fracture incidence was found to be 0.6%, and the screw loosening or fracture was often located at the first molar and maxillary central incisor restored areas [10]. However, Cervino et al. (2022) reported that all the passant screws analyzed by scanning electron microscopy after subjecting them to different screwing torques or repeating the screwing process several times were free of defects or fractures [11]. There are various systems on the market for the extraction of broken screws [12,13,14], from systems that use conventional instruments, such as probes or ultrasounds, to mechanical-based systems in screw extraction kits, which usually include guide cylinders to facilitate the removal of the broken fragment. From the large variety of systems available to the dentist, the need arises to know which is the most effective method for the extraction of broken screws. Only two in vitro studies were found that compared conventional systems with mechanical systems, and showed better results for the removal of broken fragments in implants using mechanical extraction kits [11,15]. Additionally, Nayana et al. (2022) established a treatment protocol for the management of fractured abutment screws, but focused on the fact that effort should be made to identify and eliminate the cause of screw fractures [16]. However, Raju et al. (2021) pointed out that the location of the fracture directly affects the difficulty of retrieval as well as the risk to the implant, especially the gingival location to the implant platform [17]. Moreover, Moorthy et al. (2022) reported that the time required to remove the abutment screw fragments in the maxillary arch was significantly longer than for the mandibular arch (*p* < 0.05), although the experience of the operator had no effect on the ability to successfully retrieve fractured abutment screws [18]. Additionally, Goldberg et al. (2019) evaluated and compared the removal torque value and force to failure of non-axially tightened implant abutment screws and concluded that the angulation of the abutment had no significant influence on the screw removal torque value [19]. Finally, Huang and Wang (2019) described the mechanisms and factors related to the loosening of implant abutment screws and highlighted that the internal connection and abutments with anti-rotational and conical designs have better resistance to screw loosening [20].

However, Sim et al. (2017) suggested a hollow abutment screw design to allow for the removal procedures of the fractured abutment screw and concluded that hollow abutment screws may be an alternative to conventional abutment screws [21].

The main objective of this study was to compare the effectiveness of mechanical extraction mechanisms for fractured screws in implants. The hypotheses were as follows: The mechanical extraction method would be more effective, in terms of extraction success and time required, than the conventional one. Neither method would damage the internal thread of the implants. The type of screw fracture would not influence its removal. The screws with previous mobility would be easier to extract and the experience of the operator would be important to achieve the extraction.

## 2. Materials and Method

### 2.1. Study Design

One hundred and eighty prefabricated, grade IV titanium straight abutments (E-MD-410-502 Sweden & Martina^®^, Sweden&Martina SPA, Padova, Italy) were screwed to 180 grade IV titanium implants with an internal hexagonal connection measuring 4.25 mm in diameter and 11.50 mm in length (Sweden & Martina^®^ Khono, Padova, Italy), using 180 abutment screws (VM-200 Sweden & Martina^®^, Padova, Italy). Afterwards, the abutment-implant complexes screwed with the abutment screws were randomly distributed through a free-access sample randomization web application (http://www.alazar.info, accessed on 18 January 2021) into the following removal techniques: Group A: a conventional technique using an exploration probe and an ultrasonic appliance (n = 45) (ProUltra, Dentsply Maillefer, Charlotte, NC, USA); Group B: a drilling technique using a Rhein83^®^ appliance (n = 45) (Broken Screw Extractor Kit; Rhein83^®^, Rhein83, Bologna, Italy); Group C: a drilling technique using a Sanhigia^®^ appliance (n = 45) (FSRK-01; Sanhigia^®^, Sanhigia, Saragossa, Spain); Group D: a drilling technique using a Phibo^®^ appliance (n = 45) (Phibo, Barcelona, Spain).

Subsequently, each removal technique study group was randomly (http://www.alazar.info/, accessed on 18 January 2021) according to the experience of the following operators: Operator 1: graduate student; Operator 2: general dentist; and Operator 3: dental implant specialist. The sample size was determined using a power effect of 87.2 (anything above 80 was deemed acceptable). One hundred and eighty abutment screws were included in this study to ensure a power effect of 80.00% for detecting statistically significant differences. The null hypothesis H₀: μ₁ = μ₂ was evaluated using the bilateral Student’s *t*-test of two independent samples, with a significance level of 5.00%.

### 2.2. Methodological Procedure

The implants were embedded into nylon tubes with epoxy resin (Exakto-Form^®^, Bredent GmbH & Co.KG, Senden, Germany) and angled at 30 degrees after making a cast key to standardize the position, as established in the ISO 14801 [22]. Then, the prefabricated abutments were screwed into the dental implants by applying a 30Ncm torque using a dynamometric torque wrench (Zimmer Dental^®^, Zimmer Biomet, Carlsbad, FL, USA).

#### 2.2.1. Mechanical Cycling Fatigue and Compression Load Workflow

**Figure 1:**
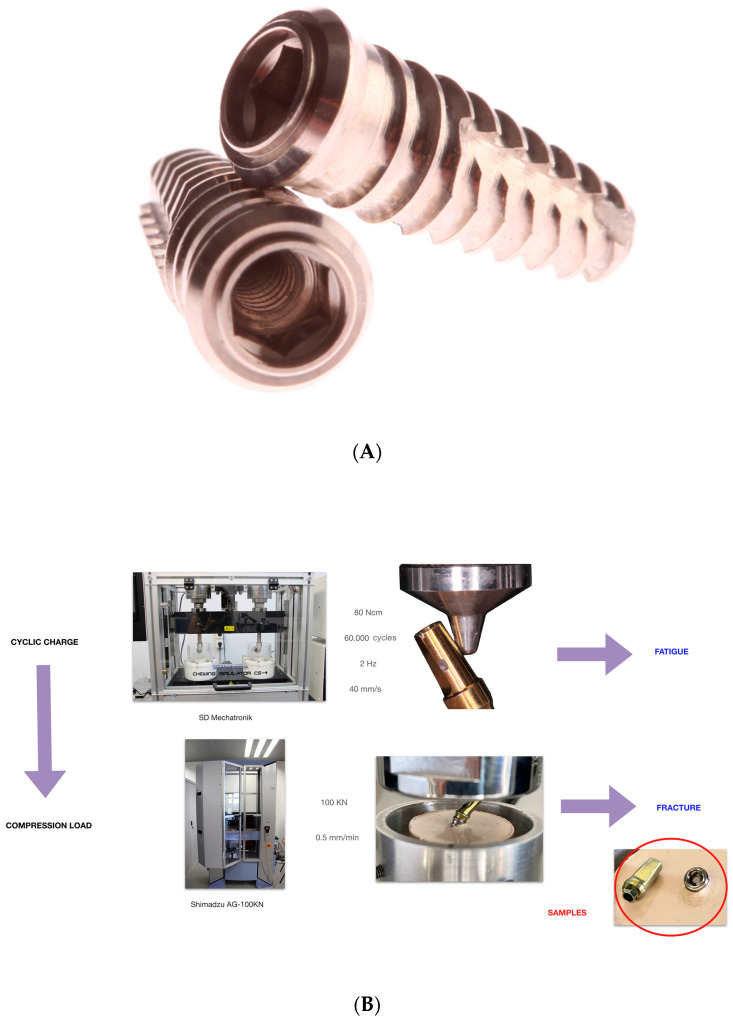
(**A**) Type of implant used in the study (Sweden & Martina^®^ Khono). (**B**) Mechanical cycling fatigue and compression load workflow to generate the fracture of the abutment screws.

The experimental samples were mechanically fatigued in a masticatory simulator (Chewing Simulator CS-4, SD Mechatronik, Rosenheim, Germany) with an applied load of 80 N for 60,000 masticatory cycles. Loading was applied at 30 degrees on the surface of the prefabricated abutments at 2 Hz frequency and 40 mm/s speed (Figure 1) [22,23,24,25,26].

#### 2.2.2. Static Load

After mechanical cycling fatigue simulation, the experimental samples were subjected to a static load until fracture of the abutment screw using a universal testing machine (UTM) (Shimadzu^®^ AG-100KN, Shimadzu Corporation, Kyoto, Japan) with a load cell of 100 KN and a crosshead speed of 0.5 mm/seg at a room temperature of 23 ± 1 °C, moving at 30 degrees on the surface of the prefabricated abutments. Axial compressive loads were exerted by sliding in a cone-shaped stainless-steel bar finished in a rounded tip (diameter: 1 mm) adapted to the UTM. This customized load piston was perpendicularly applied at the surface of the tilted prefabricated abutments until the fracture of the abutment screws, which was defined as a sharp decrease in the stress plot (Figure 1). The results were recorded using built-in software for the testing machine (PCD2K v1.0, SERVOSIS), and force (N)-displacement (mm) curves were automatically created. Additionally, the fracture level of the abutment screws was analyzed using optical microscopy (Leica M400 E microscope, L’Hospitalet de Llobregat, Spain) and classified as the coronal third, middle third, or apical third of the thread of the abutment screws.

#### 2.2.3. Conventional Removal Technique

**Figure 2:**
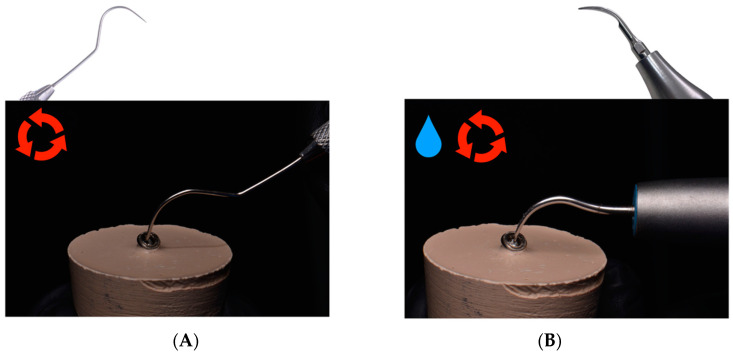
Conventional fractured abutment screw removal technique. (**A**) Exploration probe. (**B**) Ultrasound tip.

The fractured abutment screws randomly assigned to the conventional removal technique study group were removed using an exploration probe (EX23/66 Hu-Friedy, Hu-Friedy, Emmingen, Germany) (Figure 2A) and an ultrasonic tip (APU000534; Woodpecker, Guangxi, China) if necessary, engaged to an ultrasonic appliance (ProUltra, Dentsply Maillefer, Charlotte, NC, USA) with counterclockwise circular movements under irrigation, at 30 VA power and 50 Hz frequency (Figure 2B).

#### 2.2.4. Drilling Removal Technique Using Rhein83^®^ System

**Figure 3:**
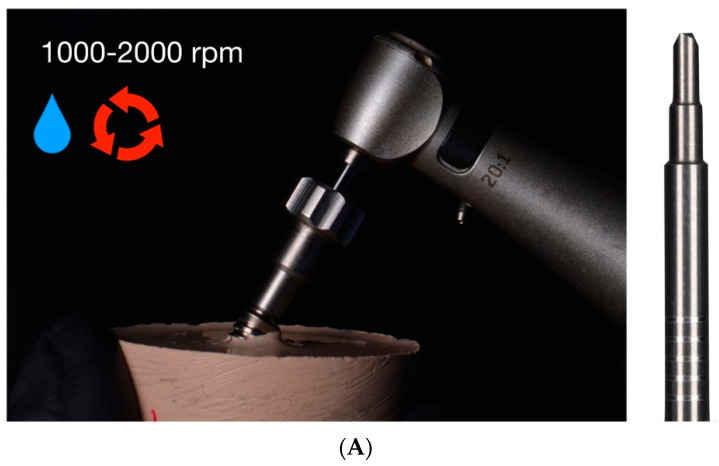

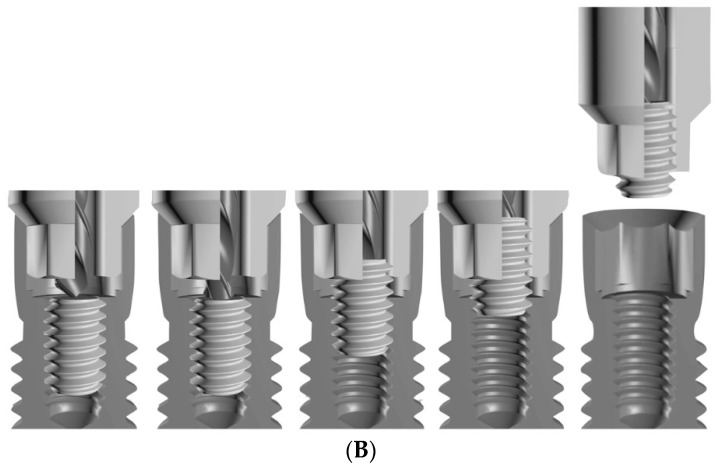
Mechanical technique of fractured abutment screw removal using the Rhein83^®^ extractor kit. (**A**) Claw reamer bur inserted through centering device fitted onto implant prosthetic platform. (**B**) Extraction protocol with claw reamer bur and centering device.

The fractured abutment screws randomly assigned to the drilling technique study group using the Rhein83^®^ appliance were removed firstly using a claw reamer bur (680FA; Rhein83^®^), which was used at 1000–2000 rpm in a 20:1 reduction counterclockwise movement with an implantology handpiece (WS-75 LG; W&H, Hawthorne Drive, MI, USA) under profuse irrigation (Figure 3A), after placing and fixing the centering device on the implant connection (Figure 3B). Subsequently, if the fractured abutment screw remained inside the dental implant, a special bur with a reverse cut was used at 500–600 rpm in a 20:1 reduction counterclockwise movement with an implantology handpiece under profuse irrigation, according to the manufacturer’s recommendations.

#### 2.2.5. Drilling Removal Technique Using Sanhigia^®^ System

**Figure 4:**
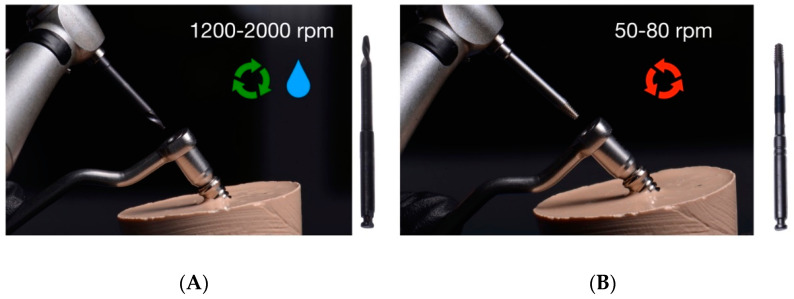
Mechanical technique of fractured abutment screw removal using the Sanhigia^®^ extractor kit. (**A**) First drill and guide fixed on the dental implant connection. (**B**) Second drill.

Additionally, the fractured abutment screws randomly assigned to the drilling technique study group using the Sanhigia^®^ appliance were removed firstly by placing and fixing the guide (TSV 3.7/4.7; Sanhigia^®^) on the implant connection. Then, the drill was used at 1200–2000 rpm in a clockwise movement with an implantology handpiece under profuse irrigation to create a perforation on the coronal surface of the fractured abutment screw (Figure 4A). Finally, a second drill was used at 50–80 rpm in a counterclockwise movement with an implantology handpiece without irrigation to remove the fractured abutment screw (Figure 4B).

#### 2.2.6. Drilling Removal Technique Using Phibo^®^ System

**Figure 5:**
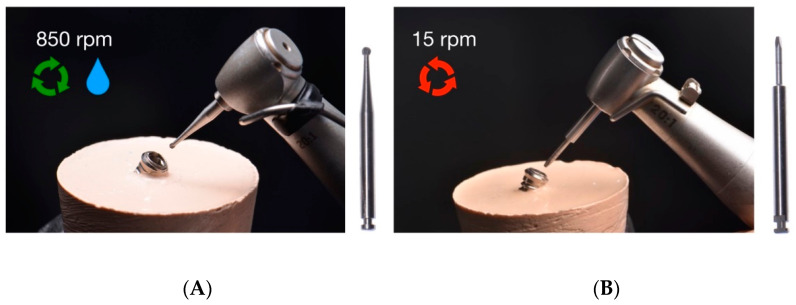
Mechanical technique of fractured abutment screw removal using the Phibo^®^ extractor kit. (**A**) Tungsten carbide round bur. (**B**) Stainless-steel pyramid-shaped drill.

Moreover, the fractured abutment screws randomly assigned to the drilling technique study group using the Phibo^®^ appliance were removed firstly using a tungsten carbide round bur at 850 rpm in a clockwise movement with an implantology handpiece under profuse irrigation to create a perforation on the coronal surface of the fractured abutment screw (Figure 5A). Afterwards, a stainless-steel pyramid-shaped drill was used at 15 rpm in a 20:1 reduction counterclockwise movement with an implantology handpiece under profuse irrigation (Figure 5B).

Finally, the time needed to remove the fractured abutment screws was recorded up to a maximum of 10 min; additionally, the internal thread of the dental implant connection was analyzed by gently screwing an abutment with an unused abutment screw. However, if the abutment screw showed resistance to be screwed, it was considered damaged.

### 2.3. Statistical Analysis

Statistical analysis of all variables was carried out using IBM SPSS Statistics, v19.0 (IBM Corporation, Armonk, NY, USA). Descriptive statistics were expressed as the mean and standard deviation (SD) for quantitative variables. An inferential analysis was carried out with the objective of studying the association between the different variables and the final result. To assess the homogeneity of the different groups of screws in each extraction method, Chi^2^-type tests were used. Simple binary logistic regression models were estimated to explain the probability of success depending on the method used and other independent variables (e.g., type of fracture, mobility, and operator). The odds ratio (OR) and 95% confidence intervals of the unadjusted association were provided. The independent variables detected as significant (*p* < 0.05) and relevant (*p* < 0.1) were used to estimate a multiple model, with which adjusted ORs were presented. The same methodology was used to study the probability of thread involvement. To study the working time in the successful subgroups according to the extraction method, a Kruskal–Wallis test was initially used and verified by the Kolmogorov–Smirnov test and Levene’s test for homogeneity of variances (*p* < 0.05). One-way ANOVA models were estimated to evaluate the mean time at the different levels of the independent factors. Finally, the relevant factors were incorporated into a multivariate ANOVA model. Two-way ANOVA models were estimated to evaluate the mean time according to the method and operator used. The level of significance used in the analyses was 5% (α = 0.05). The study had a power of 80% with a confidence level of 95%.

## 3. Results

A total of 123 out of 180 (68.3%) fractured abutment screws were successfully removed. Additionally, 51.7% (n = 93) of the abutment screws showed a fracture at the coronal third, 35% (n = 63) showed a fracture at the middle third, and 13.3% (n = 24) showed a fracture at the apical third (Table 1).

Additionally, each operator checked whether the fractured abutment screws presented mobility or not before starting the removal procedures and showed that 40% of the abutment screws presented with initial mobility, finding 72 with mobility and 108 without mobility.

The success rate of the extraction of fractured screws using conventional techniques was 71.1% and in mechanical techniques (including the three systems in the same group) was 67.4% (*p* = 0.644). This reduced success rate in mechanical methods is due to the difference in extraction efficiency between the three systems (Table 2). Statistically significant differences were shown among the removal successes of the drilling techniques (*p* = 0.002), resulting in Sanhigia^®^ as the statistically least effective drilling technique (OR = 0.36; *p* = 0.020) compared to the conventional technique, followed by the Phibo^®^ (OR = 1.00; *p* = 1.000) and Rhein83 drilling techniques (OR = 2.01; *p* = 0.133). In addition, the Rhein83^®^ drilling technique significantly increased the OR of removal success compared to the Sanhigia^®^ drilling technique (OR = 6.20; *p* < 0.001), and the Phibo^®^ drilling technique significantly increased the OR compared to the Sanhigia^®^ method (OR = 2.8; *p* = 0.020). There were no differences between the Rhein83^®^ and the Phibo^®^ drilling techniques (OR = 0.45; *p* = 0.133) for the removal success of fractured abutment screws.

Moreover, no statistically significant differences were shown related to operator experience (*p* = 0.371) or depth positioning of the fracture of the abutment screw (*p* = 0.530).

In addition, the preoperative mobility of the fractured abutment screw was a significant factor in the removal success rate, increasing the probability of removal success by twelve times compared to the fractured abutment screws without mobility (OR = 12.4; *p* < 0.001). Specifically, the removal success of the abutment screws with mobility was of 93.1% and the removal success of the abutment screws without mobility was of 51.9%. A total of 56 out of 108 abutment screws without mobility were removed from the implant. Moreover, statistically significant differences were shown among the removal techniques (*p* = 0.003); specifically, the Rhein83^®^ drilling technique removed four times more fractured abutment screws without mobility than the conventional technique (OR = 4.00; *p* = 0.021); however, the Sanhigia^®^ drilling technique reduced the probability of abutment screw removal compared to the conventional technique (OR = 0.40; *p* = 0.110), and the Phibo^®^ drilling technique showed a removal success rate similar to that of the conventional technique (OR = 0.85; *p* = 0.768). Regarding the abutment screws with mobility, 67 out of 72 were removed from the implant. Moreover, statistically significant differences were shown among the removal techniques (*p* = 0.017), resulting in the Phibo^®^ and the conventional techniques as the most successful removal techniques.

In addition, 9.8% of the dental implant connections were damaged after the extraction (*n* = 123) (Table 3). Additionally, the preoperative mobility of the fractured abutment screw was a significant factor in damaging the implant connection (OR = 0.06; *p* = 0.009), showing more damage when the fracture was located on the apical third of the implant thread (OR = 3.94; *p* = 0.097). A total of 19.6% of the fractured abutment screws without mobility showed connection damage; however, no statistically significant differences were shown between the removal techniques, although the fractures located at the apical third of the implant threads showed more damaged (*p* = 0.051). The preoperative mobility of the fractured abutment screws reduced the damage risk on the implant connection by 94%; in addition, only 1.5% of the fractured abutment screws with mobility showed connection damage. Moreover, no statistically significant differences were shown related to the operator’s experience (*p* = 0.467), or the removal technique (*p* = 0.622).

**Figure 6:**
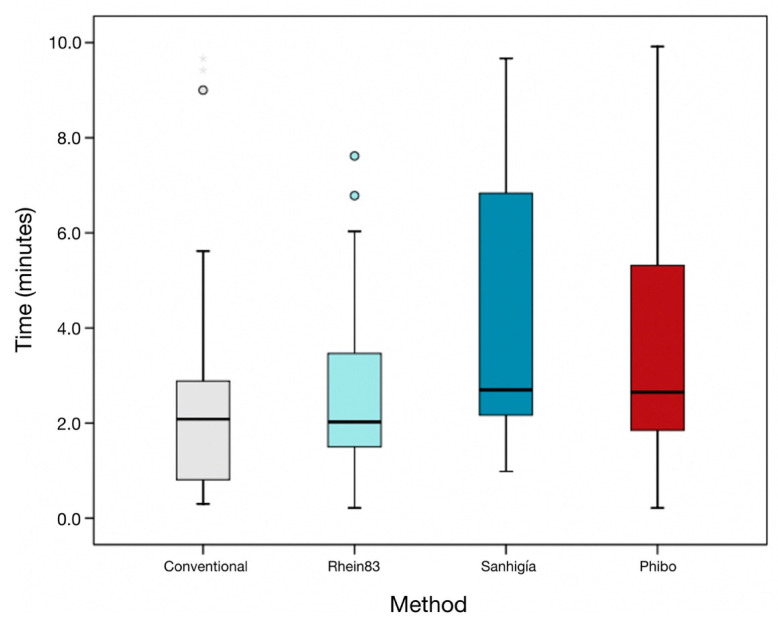
Box plot of the time required to remove the fractured abutment screws (min), according to each abutment screw removal technique. The horizontal line in each box represents the median value.

The mean and median time required to extract the fractured abutment screws was established as 3.17 ± 2.52 min, respectively (IQR = 0.81). Statistically significant differences were shown between the times required by the Sanhigia^®^ and the conventional techniques (*p =* 0.038) (Figure 6) (Table 4).

**Figure 7:**
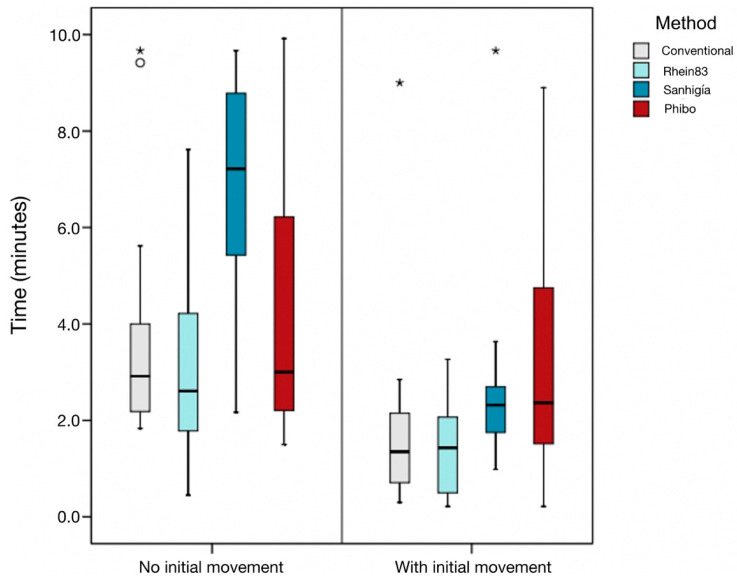
Box plot of the time required to remove the fractured abutment screws (min), depending on the mobility of the fractured abutment screw and according to each abutment screw removal technique. The horizontal line in each box represents median value.

The time required to extract the fractured abutment screws without mobility was established as 4.09 min, with the Sanhigia^®^ drilling technique requiring a statistically significant higher time (*p* = 0.006). The preoperative mobility reduced the working time to 2.39 min. Statistically significant differences were not shown between the times required by the drilling techniques (Figure 7). There were no significant differences found in the mean times for each operator in any type of screw (*p* = 0.328 without and *p* = 0.092 with mobility). 

**Figure 8:**
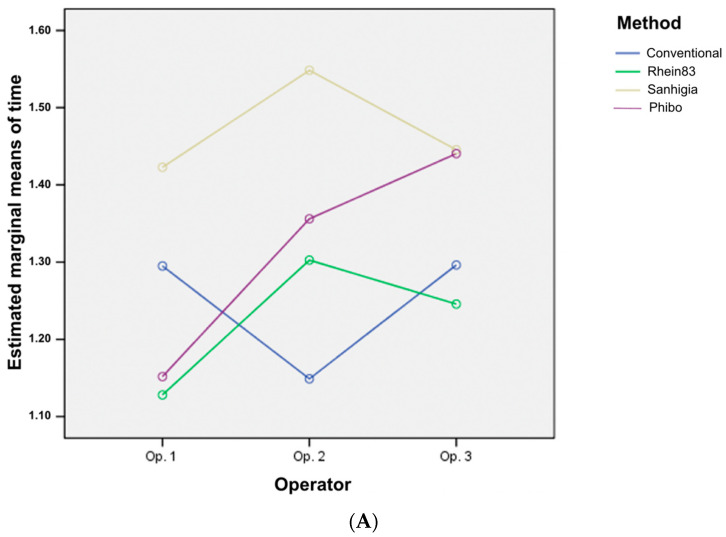

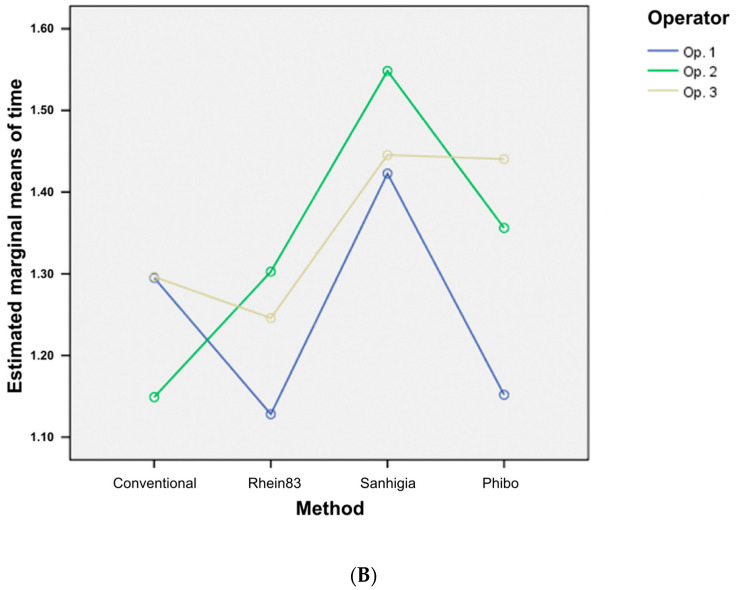
Two-way ANOVA analysis depending on the time/operator (**A**) and according to the time/method (**B**). The time with the Sanhigia method is clearly superior for any operator. For operator 1, there are significant differences between the Sanhigia and Rhein techniques (*p* = 0.031).

In immobile screws, the extraction time depends fundamentally on the extraction method (*p* = 0.006). There is a trend in relation to the operator (*p* = 0.092), but it can be stated that the differences in times depending on the method can be extended to any operator (*p* = 0.228) (Figure 8).

## 4. Discussion

The results obtained in the present study rejected the null hypothesis (H_0_) that states that there will be no difference between the removal capability, dental implant connection damage, and time required to remove the fractured abutment screws between three drilling techniques and a conventional method using an exploration probe and ultrasonic appliance for the extraction of fractured abutment screws. The Rhein83^®^ method obtained the highest removal rate (84.4%); however, Agustín-Panadero et al. obtained higher results (96.7%) [15]. These results were followed by the Phibo^®^ drilling technique, with a removal rate of 71.7%; however, Agustín-Panadero et al. obtained a success rate of 93.3% [12]. Lastly, the Sanhigia^®^ drilling technique showed a success rate of 46.7%, although Agustín-Panadero et al. obtained a lower rate (20%) [15]. As a result, the authors observed that the extraction of fixed screws requires overcoming the screw torque with the drilling technique; however, manual removal of screws with mobility is safer than using drilling techniques. The removal technique using the Rhein83^®^ system is based on a claw reamer bur which does not need specific drilling, since this bur can remove the fractured abutment screw by engaging the coronal surface of the fragment in a counterclockwise movement. Therefore, it reduces the number of instruments and procedures, reducing the risk of complications. However, the removal techniques using the Sanhigia^®^ and Phibo^®^ systems require a guided drilling procedure which may increase the temperature despite irrigation, leading to an expansion of the metals that can increase the resistance between the fractured abutment screws and the dental implant connection, making them difficult to remove. Additionally, Brisman et al. highlighted that the drilling time directly affects the temperature, and the Sanhigia^®^ and Phibo^®^ systems required more time than the Rhein83^®^ system [16]. In addition, Ercoli et al. suggested that the sharpness of the drills may significantly affect the cutting efficiency and heat generation of the drills. Hence, the claw reamer bur presents a geometrical design which promotes the engagement of the fractured abutment screws without drilling [17].

After extracting the screws, the integrity of the internal thread of the implant connection was assessed, observing a damage rate of 9.8%, to the point of preventing the subsequent placement of the prosthesis. However, Agustín-Panadero et al. observed a 13.33% [12] and 7.8% [15] involvement of the internal thread of the implant connection. However, the previous mobility of the abutment screw is a determining factor in its removal, and therefore, in the probability of causing damage to the internal thread of the implant connection, reducing the probability of damage by 94%. In addition, the height of the abutment screw fracture was also a relevant factor, resulting in the apical fragments being more susceptible to damaging the connection. Fortunately, abutment screws fracture most frequently at the coronal level (51.7%), followed by the midlevel (35%), and the apical level (13.3%). This may be because the junction between the body and the threads of the abutment screw is at the coronal level. In addition, Agustín et al. observed a very similar screw fracture pattern, with coronal fractures being the most frequent (58.3%), followed by median (28.3%) and apical (13.3%) fractures. The Phibo^®^ drilling technique may increase the probability of damaging the internal thread of the implant connection due to the absence of a guide.

Regarding the mechanical or specific extraction systems, they achieve better results in the recovery of the screw fragments, recovery time, and preservation of the internal thread of the implants [18,19,20,21,22,23,24,25,26,27,28,29]. The conventional method in which a probe and ultrasound are used is efficient as well as economical; therefore, it is a good method for the extraction of fractured abutment screws and this is supported by the statistical data found in the different studies that have a 73.3% extraction success with this method [12,15]. In our research, an extraction success rate for the conventional method of 71.1% was found. Finally, the extraction rate in the mobile abutment screws was 93.1% and in the fixed abutment screws was 51.9%; however, Agustín-Panadero et al. did not differentiate whether the fragment was mobile or not [15]. In addition, the results of the present study do not agree with those obtained by Bufalá Pérez et al., since they reported that the drilling technique without irrigation provides a lesser removal capability, less conical internal hex implant-abutment connection damage, and less thermal effect than the ultrasonic technique for the extraction of fractured abutment screws; however, the ultrasonic technique was more effective for the extraction of fractured abutment screws [29].

The experimental model of this laboratory-based study can be easily transferred to a clinical setting, since all the methodology procedures were performed according to the manufacturer’s recommendations; moreover, this methodological design was validated in previous studies [12,15].

## 5. Conclusions

Based on the results of this study and taking into account its limitations, it can be concluded that the Rhein83^®^ drilling technique showed the highest removal rate of fractured abutment screws, although the previous mobility of the fractured abutment screws resulted decisive, causing a higher removal rate, a shorter removal time and less damage to the internal thread of the implant connection.

## Figures and Tables

**Table 1:** Type of fracture of screws used in this research according to the extraction method applied (sample randomization).

	Method									
	Total		Conventional		Rhein83		Sanhigia		Phibo	
	N	%	N	%	N	%	N	%	N	%
**Total**	180	100.0%	45	100.0%	45	100.0%	45	100.0%	45	100.0%
**Coronal**	93	51.7%	23	51.1%	25	55.6%	26	57.8%	19	42.2%
**Half**	63	35.0%	15	33.3%	13	28.9%	15	33.3%	20	44.4%
**Apical**	24	13.3%	7	15.6%	7	15.6%	4	8.9%	6	13.3%

**Table 2:** Success of extraction of the fractured fragment within the implant according to the operator and method used.

					Total	No	Yes
**Method**	**Total**	**Operator**	**Total**	**N**	180	57	123
				**%**	100.0%	31.7%	68.3%
			**Op. 1**	**N**	60	20	40
				**%**	100.0%	33.3%	66.7%
			**Op. 2**	**N**	60	22	38
				**%**	100.0%	36.7%	63.3%
			**Op. 3**	**N**	60	15	45
				**%**	100.0%	25.0%	75.0%
	**Conventional**	**Operator**	**Total**	**N**	45	13	32
				**%**	100.0%	28.9%	71.1%
			**Op. 1**	**N**	15	3	12
				**%**	100.0%	20.0%	80.0%
			**Op. 2**	**N**	15	6	9
				**%**	100.0%	40.0%	60.0%
			**Op. 3**	**N**	15	4	11
				**%**	100.0%	26.7%	73.3%
	**Rhein83**	**Operator**	**Total**	**N**	45	7	38
				**%**	100.0%	15.6%	84.4%
			**Op. 1**	**N**	15	4	11
				**%**	100.0%	26.7%	73.3%
			**Op. 2**	**N**	15	3	12
				**%**	100.0%	20.0%	80.0%
			**Op. 3**	**N**	15	0	15
				**%**	100.0%	0.0%	100.0%
	**Sanhigia**	**Operator**	**Total**	**N**	45	24	21
				**%**	100.0%	53.3%	46.7%
			**Op. 1**	**N**	15	9	6
				**%**	100.0%	60.0%	40.0%
			**Op. 2**	**N**	15	8	7
				**%**	100.0%	53.3%	46.7%
			**Op. 3**	**N**	15	7	8
				**%**	100.0%	46.7%	53.3%
	**Phibo**	**Operator**	**Total**	**N**	45	13	32
				**%**	100.0%	28.9%	71.1%
			**Op. 1**	**N**	15	4	11
				**%**	100.0%	26.7%	73.3%
			**Op. 2**	**N**	15	5	10
				**%**	100.0%	33.3%	66.7%
			**Op. 3**	**N**	15	4	11
				**%**	100.0%	26.7%	73.3%

**Table 3:** Internal thread impact of the implant according to method used.

	Method									
	Total		Conventional		Rhein83		Sanhigia		Phibo	
	N	%	N	%	N	%	N	%	N	%
**Total**	123	100.0%	32	100.0%	38	100.0%	21	100.0%	32	100.0%
**No**	111	90.2%	27	84.4%	35	92.1%	19	90.5%	30	93.8%
**Yes**	12	9.8%	5	15.6%	3	7.9%	2	9.5%	2	6.3%

**Table 4:** Extraction time (minutes) depending on the operator per method.

	Method																			
	Total				Conventional				Rhein83				Sanhigia				Phibo			
	Operator				Operator				Operator				Operator				Operator			
	Total	Op. 1	Op. 2	Op. 3	Total	Op. 1	Op. 2	Op. 3	Total	Op. 1	Op. 2	Op. 3	Total	Op. 1	Op. 2	Op. 3	Total	Op. 1	Op. 2	Op. 3
**N**	123	40	38	45	32	12	9	11	38	11	12	15	21	6	7	8	32	11	10	11
**Average**	3.17	2.73	3.17	3.55	2.58	3.48	0.99	2.90	2.57	1.72	3.08	2.78	4.32	4.09	4.10	4.68	3.70	2.19	4.58	4.41
**Standard** **deviation**	2.52	2.18	2.70	2.65	2.50	2.97	0.72	2.42	1.78	1.21	2.25	1.57	3.04	2.23	3.58	3.39	2.67	1.27	2.65	3.22
**Minimo**	0.22	0.22	0.30	0.22	0.30	0.68	0.30	0.73	0.22	0.22	0.50	0.43	0.98	1.47	1.00	0.98	0.22	1.25	1.98	0.22
**Maximum**	9.92	9.42	9.92	9.67	9.67	9.42	2.18	9.67	7.62	4.27	7.62	6.03	9.67	6.83	9.67	9.67	9.92	5.88	9.92	8.90
**25th** **percentile**	1.50	1.49	1.38	1.98	0.81	1.70	0.40	2.08	1.50	0.50	1.55	1.53	2.17	1.75	2.00	2.25	1.85	1.50	2.93	1.12
**Median**	2.22	2.01	2.30	2.50	2.08	2.47	0.78	2.23	2.03	1.62	2.61	2.22	2.70	4.13	2.32	3.02	2.65	1.92	3.36	4.27
**75th** **percentile**	4.17	3.16	3.95	4.33	2.88	4.30	1.38	2.98	3.47	2.07	4.06	3.47	6.83	6.23	8.90	8.13	5.32	2.22	6.00	7.07

## Data Availability

Information is available on request in accordance with any relevant restrictions (e.g., privacy or ethics).
